# Preparing for Severe Acute Respiratory Syndrome Coronavirus 2 (SARS-CoV-2) Self-Testing Implementation: Lessons Learned From HIV Self-Testing

**DOI:** 10.3389/fmed.2020.599521

**Published:** 2020-12-07

**Authors:** Donaldson F. Conserve, Allison Mathews, Augustine T. Choko, LaRon E. Nelson

**Affiliations:** ^1^Department of Prevention and Community Health, Milken Institute School of Public Health, George Washington University, Washington, DC, United States; ^2^Department of Health Behavior, Gillings School of Global Public Health, University of North Carolina at Chapel Hill, Chapel Hill, NC, United States; ^3^Maya Angelou Center for Health Equity, Wake Forest Baptist Medical Center, Winston-Salem, NC, United States; ^4^Malawi-Liverpool Wellcome Trust Clinical Research Programme, Blantyre, Malawi; ^5^Yale School of Nursing, Orange, CA, United States; ^6^Unity Health Toronto, St. Michael's Hospital, MAP Centre for Urban Health Solutions, Toronto, ON, Canada

**Keywords:** HIV - human immunodeficiency virus, SARS-CoV-2, self-testing, implementation science framework, COVID-19, COVID-19 testing

## Introduction

The number of severe acute respiratory syndrome coronavirus 2 (SARS-CoV-2) cases and associated death continue to rise globally. Widespread testing for SARS-CoV-2 infection is crucial in order to identify individuals who need to need to be isolated, thereby reducing their chances to infect others and allowing them to seek treatment earlier which can prevent further negative health outcomes and mortality (1). Currently, the most common testing method for SARS-CoV-2 diagnosis is Real-Time Polymerase Chain Reaction (RT-PCR) from nasopharyngeal, throat or saliva specimens (2). However, SARS-CoV-2 testing has been hampered in many countries due to inadequate test kits, uncomfortable testing procedures, shortages of personal protective equipment (PPE) for health care workers, and low demand among people to seek testing for SARS-CoV-2 at health facilities (3–7).

In response, the United States Food and Drug Administration (FDA) provided Emergency Authorization Use for several SARS-CoV-2 self-sampling kits (SARS-CoV-2SS) that allow individuals to self-collect nasal swabs and saliva specimens and send to a lab for testing (8). Other efforts to increase testing include drive-through methods that include both self-sampling and health care- collected samples (9, 10). The National Institute of Health has also launched the Rapid Acceleration of Diagnostics (RADx) program to accelerate the development of, scale up, and deploy innovative point-of-care of technologies, support the scale-up of more advanced technologies, and nontraditional approaches for testing as well as establish community-engaged implementation projects to improve access to testing in underserved and vulnerable populations (11). Similar research and programmatic activities to increase testing capacity SARS-CoV-2 are also being implemented in other regions (12–16).

The efforts to increase testing capacity for SARS-CoV-2 diagnosis testing will be enhanced with the availability and widespread promotion of self-sampling and eventually SARS-CoV-2 self-testing (SARS-CoV-2ST) (17–19). The benefits of self-sampling and self-testing include their abilities to help decentralize care, promote social distancing, conserve PPEs, address transportation and privacy barriers for individuals who do not want to test at a clinic or a drive-through setting, and reach more individuals who are not reached with current testing modalities (12, 17, 20). Unlike self-sampling, SARS-CoV-2ST will allow individuals to receive their results at home without the need to ship their specimens to a laboratory for testing (21, 22). To our knowledge, there is only one FDA-approved SARS-CoV-2ST diagnostic kit for individuals to use and receive their results at home as of November, 17, 2020 (23). However, a number of other different at-home SARS-CoV-2ST kits are being developed and evaluated to either detect antibodies or active viral infections (24). Antibody SARS-CoV-2ST kits reveal markers of immune response that show up in blood more than a week after a person has been infected whereas active infections will be detected with nucleic acid SARS-CoV-2ST kits through the virus' genetic materials (24, 25).

As researchers, federal health agencies, and public health practitioners prepare to implement SARS-CoV-2ST, lessons learned from the global implementation and scaling up efforts for HIV self-testing (HIVST) can prove useful. We describe research related to questions that emerged regarding HIVST and how they are similar or different to the questions that will need to be addressed for SARS-CoV-2ST before this crucial strategy can be implemented and scaled up successfully. We also discuss the findings of the first antibody SARS-CoV-2ST acceptability and usability study (22) and the public health implications of and recommendations people who obtain a positive SARS-CoV-2ST result for antibodies or active viral infections. Lastly, we identify key structural inequities in communities that are most affected by COVID-19 that need to be addressed during future SARS-CoV-2ST implementation efforts.

## In-Home HIV Self-Testing History

In 2012, the US FDA approved the first over-the-counter rapid HIVST kit, the OraQuick In-Home HIV Test which allows users to test for antibodies using saliva sample, similar to the new saliva-based SARS-CoV-2ST kit (26), and receive a preliminary result at home in 20 min (27). The benefits of HIVST include privacy, an increase of access to HIV testing, earlier diagnosis of HIV, confidentiality of results, and reducing queues for facility-based HIV testing (28). HIVST can also help bypass social barriers such as stigma and discrimination that deter people from accessing facility-based HIV testing (28). Since the approval of HIVST, several questions emerged about its accuracy, acceptability, feasibility, the lack of pre-and-post-test counseling, whether users would seek a confirmatory test, and link to care (29). There is now overwhelming evidence that HIVST is accurate, acceptable, feasible, and effective with minimal social harms (29). As a result from these studies, the World Health Organization (WHO) now recommends HIVST as one of the testing strategies for HIV prevention efforts (30). These studies, including our own (31–39), have provided evidence on different distribution strategies from online platforms, peers to sexual partners, community health workers (40–42). Similarly, these studies have assessed different approaches to verify HIVST results either through direct supervision by health provider, requesting participants to return used HIVST kits, electronic transmission of photographs, or using Bluetooth sensors (43).

## Antibody SARS-CoV-2 Self-Testing Acceptability and Usability

In the first published SARS-CoV-2ST study, researchers in England examined the acceptability and feasibility of two types (i.e., Guangzhou Wondfo Biotech Co Ltd and Fortress Orient Gene Biotech Co Ltd) of SARS-CoV-2ST lateral flow immunoassays (LFIAs) or rapid point-of-care tests that use a blood sample from a finger-prick and produce a self-read result after 10 or 15 min for detection of SARS-CoV-2 antibodies (Immunoglobin M and Immunoglobin G) (22). Participants received LFIAs by mail and recorded their interpretation of their results in an online survey with the option to upload a photograph of the results (22). To assess participants' ability to correctly interpret the test results, a clinician reviewed all the samples of the uploaded photographs that were reported as positive and unable to read as well as a random sample of 200 participant-reported negative or invalid results. Acceptability in the national study was high with 99.3% (8,693/8,754) and 98.4% (2,911/2,957) of participants reporting that they attempted to use the two LFIA types (22). Feasibility was also high in the pilot and national studies with 86.5% (225/260) of pilot participants and 97.5 and 97.8% of participants in the national study reporting they completed all the steps for the tests successfully, respectively (22). The majority of participants 85.8% (7,272/8,475) and 84.8% (2,416/2,848) uploaded the photographs of their results with substantial agreement between participant and clinician interpreted results for both test types (22). However, there were differences between some of the self-reported results and those reported by the clinician and some participants reported some difficulties with using the lancet and pipette of the test kits (22).

## Discussion

The scientific and clinical fields involved in HIV prevention have provided extensive experience, amassed over decades, regarding the value of testing and the added benefits of in-home self-testing (30). This experience can be brought to bear for SARS-CoV-2ST, including strategies that can help avoid repeating the pitfalls encountered during the path toward implementing and scaling up HIVST. For example, limited evidence on the public health impact and cost-effectiveness of HIVST, uncertain levels of consumer demand and concerns about potential social harms amongst others delayed the roll out of HIVST (44). Global efforts and collaborations between WHO, researchers, local health agencies, donors, and policy makers have addressed some of these limitations. Initiatives such as but not limited to the Self-**T**esting **A**f**R**ica (STAR), the largest HIVST implementation science project to date (44), 4 Youth by Youth crowdsourced HIVST interventions (45, 46), and **S**elf-**T**esting **E**ducation and **P**romotion (STEP) project (28, 33), have created a market for HIVST in sub-Saharan Africa. These initiatives combined with other studies around the globe have accelerated access to HIVST by gathering the necessary acceptability, feasibility, and fidelity data, creating an enabling environment with regards to HIVST policies, generating diverse demand through multiple distribution channels, and creating advocacy for additional financing, as well as accelerate market entry for suppliers at affordable and sustainable prices (44, 45, 47–49). Similar initiatives are needed swiftly to gather additional accuracy, acceptability, feasibility, and programmatic data to encourage policy makers, donors, and local health agencies to support for SARS-CoV-2ST implementation and scale up.

While the findings from the first antibody SARS-CoV-2ST acceptability and usability study in England were promising, some participants reported difficulties using the pipette and applying the blood drop to the cassette (22). Thus, more studies are needed to assess ease of use of SARS-CoV-2ST and how to provide the support that potential users may need. One potential strategy to support SARS-Co-V-2ST users is online real-time instructions, which has been evaluated with HIVST and found to be acceptable and successful in increasing HIV testing (50). A recent SARS-CoV-2SS study has shown that participants are willing to self-collect specimens [saliva, oropharyngeal swab (OPS), and dried blood spot (DBS) card] at home while being observed by a clinician through a telehealth session (51). A total of 159 participants were mailed kits and 153 scheduled a video appointment with the majority of the (*n* = 143) completing all three self-collected samples (52). A similar approach can be assessed for SARS-CoV-2ST to move beyond simply observing potential users to providing additional instructions and post-test supports in the self-testers receive a positive result.

The public health implications of potential positive antibody SARS-CoV-2ST results extend beyond treatment since individuals with antibodies for SARS-CoV-2 are considered to have recovered from COVID-19 and should less symptomatic. However, a positive antibody SARS-CoV-2ST result will allow individuals, including skeptics, to learn indeed whether they had a COVID-19 infection—increasing their perception of risk and potentially positively influencing future behaviors to prevent COVID-19 re-infection. Alternatively, a positive antibody SARS-CoV-2ST result has the potential to help individuals make informed decisions about their risk levels as they consider returning to work or interact with infected individuals (19). In addition, antibody SARS-CoV-2ST results can help identify qualified individuals who may be interested in donating blood for convalescent plasmaexternal icon as a treatment for COVID-19. The Centers for Disease Control and Prevention (CDC) describe general recommendations for positive antibody test results that people who receive a positive antibody SARS-CoV-2ST result can follow such as continuing with normal activities, washing hands often, avoiding close contact, wearing a mask when around others, and continued use of PPE if the person is a health care worker or first responder (53, 54). On the other hand, a nucleic acid SARS-Co-V2ST kit will provide individuals with active infections an instant preliminary positive result that can allow them to follow recommendations for people who are sick such as self-isolate in order to prevent potential transmission and seek confirmatory diagnostic testing and early treatment (19).

We must also be mindful that the SARS-CoV-2 transmission profile is not the same as HIV and it presents immense new challenges that will require us to envision and test new ways for its easy and reliable detection and its equitable access among marginalized racial groups, sexual minority ages, incomes and the multitude of intersections between them. As is the case with HIV, Black communities are disproportionately affected by COVID-19 (55). This population has experienced extensive barriers to facility-based HIV testing and HIVST (35) and the pattern seems to be repeating for COVID-19. We need novel ways to ensure the most vulnerable populations, who are also the most likely to be infected with and die from COVID-19, have access to affordable SARS-CoV-2ST. It is important for investigators who are validating SARS-CoV-2ST kits in community settings to design the studies in a way that ensures adequate representation from the populations most vulnerable to COVID-19 infection and mortality. To promote adequate representation of special populations (elderly aged 65 years and older, youth aged 17 years and younger, Black, Latinx, Tribal communities indigenous to North America, and Spanish-speaking and francophone populations) in SARS-CoV-2ST research and programs, there are several lessons learned from HIV research and HIVST that can be applied to COVID-19.

## Author Contributions

DFC conceived the idea for the manuscript and drafted it before all authors reviewed and edited the manuscript into this final form. All authors contributed to the article and approved the submitted version.

## Conflict of Interest

The authors declare that the research was conducted in the absence of any commercial or financial relationships that could be construed as a potential conflict of interest.
